# Versatile and High-throughput Force Measurement Platform for Dorsal Cell Mechanics

**DOI:** 10.1038/s41598-019-49592-1

**Published:** 2019-09-16

**Authors:** Seungman Park, Yoon Ki Joo, Yun Chen

**Affiliations:** 1 Department of Mechanical Engineering, Johns Hopkins University, Baltimore, MD USA; 2 Department of Chemical and Biomolecular Engineering, Johns Hopkins University, Baltimore, MD USA; 3 Center for Cell Dynamics, Johns Hopkins University, Baltimore, MD USA; 4 Institute for NanoBio Technology, Johns Hopkins University, Baltimore, MD USA

**Keywords:** Biophysical methods, Lab-on-a-chip

## Abstract

We present a high-throughput microfluidics technique facilitating *in situ* measurements of cell mechanics parameters at the dorsal side of the cell, including molecular binding strengths, local traction forces, and viscoelastic properties. By adjusting the flow rate, the force magnitude exerted on the cell can be modulated ranging from ~14 pN to 2 nN to perturb various force-dependent processees in cells. Time-lapse images were acquired to record events due to such perturbation. The values of various mechanical parameters are subsequently obtained by single particle tracking. Up to 50 events can be measured simultaneously in a single experiment. Integrating the microfluidic techniques with the analytic framework established in computational fluid dynamics, our method is physiologically relevant, reliable, economic and efficient.

## Introduction

**Figure 1:**
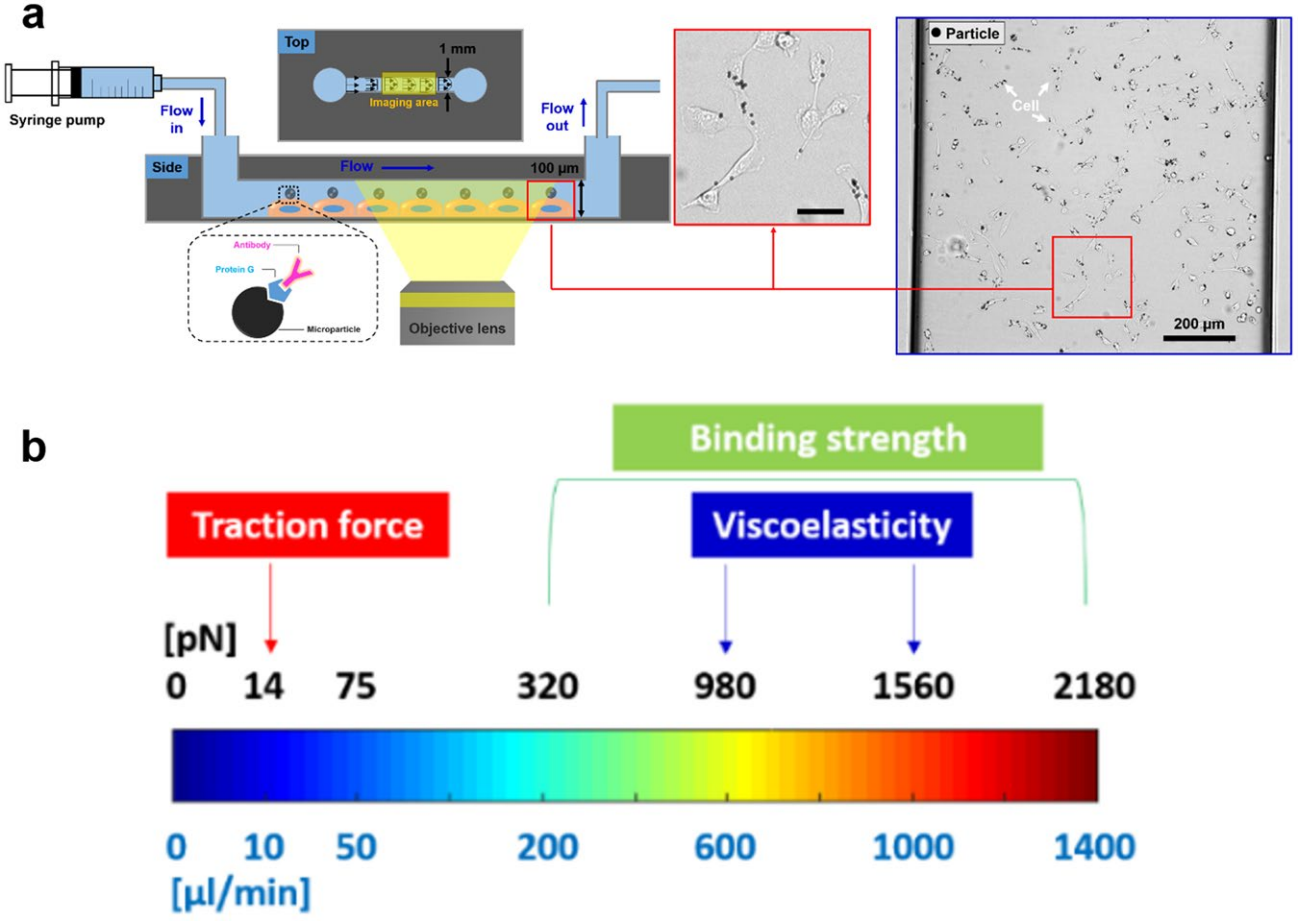
The traction force, viscoelastic properties, and bond strength of the dorsal side of cells were evaluated using the microfluidic device at high-throughput. (**a**) Cells are seeded in a microfluidic channel and then particles coated with ligand targeting surface molecules are added to bind to the cell surface. To induce hydrodynamic forces, a syringe pump and tubing are used. Particle-bound cells are shown in the inset marked (red box, Scale bar: 50 μm). Whole field of view of actual image taken for highthroughput analysis was shown (blue box). Black dots indicate particles attached to cells (white arrows). (**b**) Force spectrum is shown as a function of flow rate. Different flow rates can be applied for various types of measurement. For example, to measure the traction force, a force in the range of tens of pN is used. To measure the viscoelastic properties, forces of in the range of 1 nN are used. To measure the bond strength, a varied force which increases in a stepwise manner is used, ranging from ~300 pN to ~2000 pN.

Force measurement studies have quantitatively informed the interactions between binding partners at the molecular level, the orchestrated force-generating machinery such as cell migration and endocytosis, at the organelle level, and the mechanical properties of the cells at the cellular level^1–3^. A host of techniques has been developed to serve these purposes depending on the length scale. For example, optical trap^4,5^, atomic force microscopy (AFM)^6,7^, DNA-based force sensors^8–10^, are commonly used to measure the binding strength between two species of molecules; traction force microscopy (TFM)^11,12^ and microposts^13,14^ are commonly used to measure the contractile forces generated by cells; magnetic tweezers^15,16^ and micropipette aspiration^17,18^ are widely used to measure the viscoelastic properties of single cells. Most of the force measurement techniques described above are used in a narrow range of length scales, either at the molecular level, the organelle level or the cellular level. In addition, the techniques are low-throughput and can only measure one or a few test objects at a time. In particular, AFM and optical traps had been used to probe the mechanical properties of the cells, but the their relatively low throughput capacity results in time-consuming measurements to survey sample populations to achieve statistical significance, especially in the case when there is high heterogeneity in the sample population. Here we developed a new microfluidic-based platform and corresponding technique, which is versatile to measure forces across multiple length scales. By computing the hydrodynamic forces based on the acquired images, our new technique can measure molecular binding strengths, local traction forces, and viscoelastic properties of the cell. Our technique is relatively low-cost compared to others, requiring a syringe pump and flow chambers (Fig. 1a). By adjusting the flow rate, the applied force magnitude can be modulated within a wide range, from approximately 14 pN to 2 nN (Fig. 1b), suitable for measuring many force-mediated processes. The experiments are recorded using time-lapse bright-field microscopy at low magnification (10X), which provides a large field of view, thus high-throughput readouts, measuring up to 50 events at a time.

## Results and Discussion

We first set out to demonstrate that we could measure traction forces at the dorsal side of the cell. It should be noted that while there are multiple well-established methods to measure traction forces at the ventral side of the cell^19,20^, including traction force microscopy (TFM) and micropost assays, few methods exist to measure dorsal traction forces^21^. However, dorsal traction forces are of biological importance as well. For example, dorsal traction forces are involved in disrupting cancer cell-endothelium association during intravasation^22,23^, or in cell motility of neural crest during embryonic development^24–26^, or during the interaction between the cancer cells and immune cells^18,27–29^ which might result in immunosuppression. Here we used our platform to examine the force transmitted to the key immunosuppressing molecule cytotoxic T-lymphocyte-associated protein 4 (CTLA4)^30–34^ on the surface of cancer cells. It has been reported CTLA4 was involved in mechanically engaging its ligands CD80 or CD86 leading to immunosuppression^28,30,35,36^, though the magnitude of the mechanical forces transmitted via CTLA4 is yet to be quantified. We tracked the displacement of micron-sized particles conjugated with CD80, which bound to CTLA4^37^ on the surface of breast cancer cells (MDA-MB-231). Based on the tracking results, we calculated the magnitude of the traction force generated by the cells to overcome the hydrodynamic forces and subsequently move the particle in the direction opposite to the flow. In our system, the bond between CD80 and CTLA4 was mechanically engaged when CD80-coated particles were subjected to low hydrodynamic forces at the flow rate of 10 µl/ml, the hydrodynamic forces provided by the flow forms strong catch bonds^38,39^, enabling CTLA4 linked to the actomyosin machinery^40^ to withstand the tension while being displaced opposite to the flow direction. Therefore, the traction forces were then transmitted effectively, evident by the observation of particles being displaced in the opposite direction to the flow (Supplementary Movie S1). It should be noted that without the low hydrodynamic forces supplied by the flow, the catch bond was not activated, and no traction forces between CD80 and CTLA4 could be detected (data not shown).

**Figure 2:**
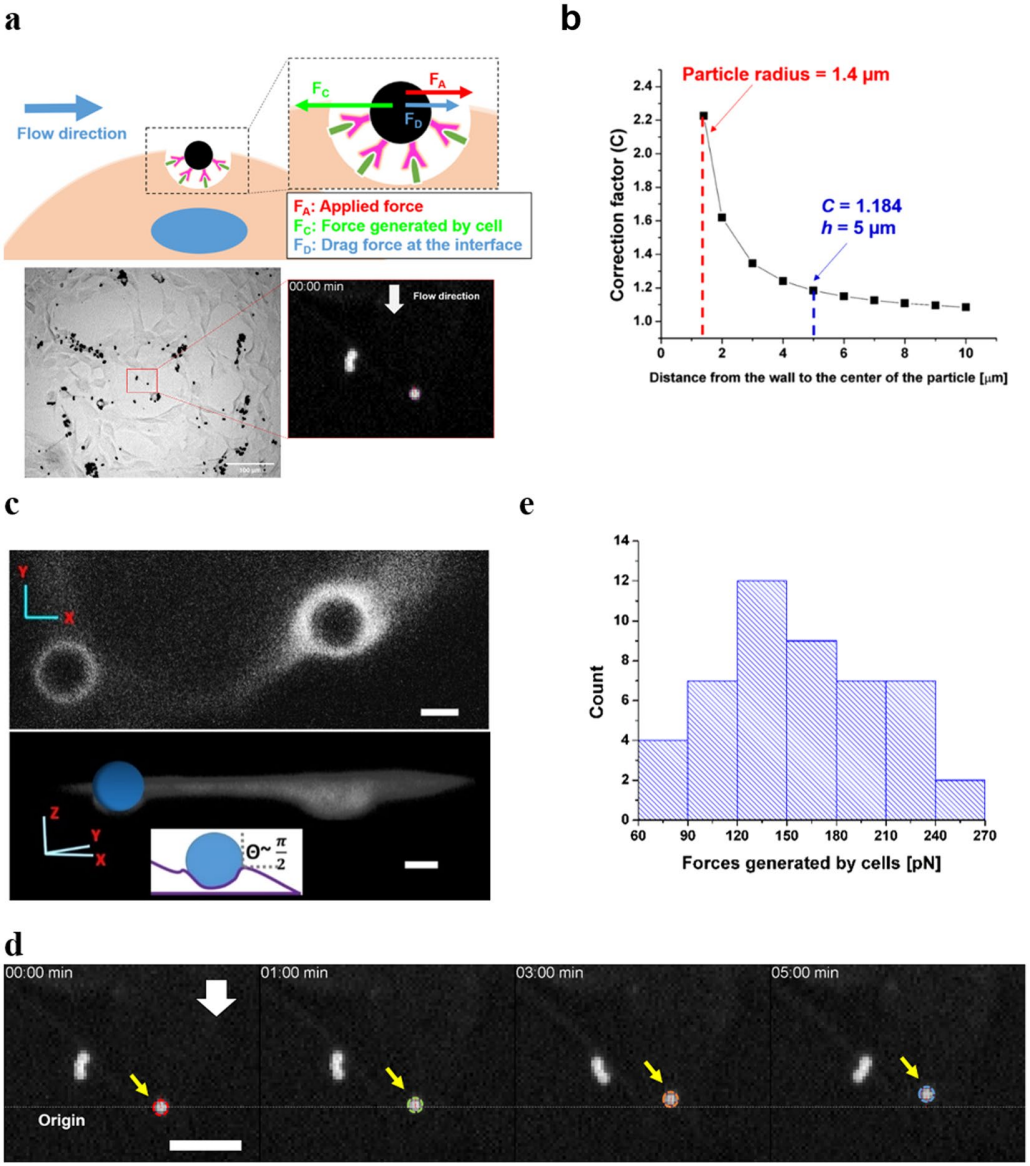
The traction forces on dorsal side of cells can be measured. (**a**) The dynamic displacement of the particle is driven by the forces generated by the cell to overcome the applied hydrodynamic force and drag force at the interface. In other words, the force generated by the cells is the vector sum of the applied hydrodynamic force and the drag force at the interface between the cell and the fluid. Please note that the original movies (Supplementary Movie S1) were inverted so that the particle appears to be bright and the background dark, for the purpose of better visualization. (**b**) The correction factor for the drag force near surfaces based on the computational simulation. (**c**) A representative x-y image (top) and 3D reconstruction image (bottom) show that the particles are around 50% enveloped by the cell membrane, forming 90° contact angle (inset) between the particle, the cell and medium. The membrane surrounding the particles appears to be brighter because the membrane is folded to accommodate the cell-bound particle. Scale bar: 2 µm. (**d**) A cell-bound particle is displaced when the flow is on at 10 µl/min and moves in the opposite direction to the flow. The flow was turned on 10 seconds before the timestamp 00:00 min. White arrow indicates flow direction. Scale bar: 20 μm. (**e**) 2.8-µm particles conjugated with CD80 on MDA-MB-231 cells were tracked, and the magnitude distribution of the force generated by the cells are shown in the histogram with average ± standard error of 157.6 pN ± 7.2 pN and median of 154.2 pN (n = 48).

The traction forces were estimated using the equation

1 $${F}_{C}={F}_{A}+{F}_{D}$$

where F_C_, F_A_, and F_D_ are the traction force generated by cell, applied hydrodynamic force, and drag forces at the interface, respectively (Fig. 2a). To estimate the hydrodynamic forces caused by the flow, calculation based on the Stokes equation was used. Stokes equation is applicable to objects moving uniform flow under laminar flow^41^, and the maximum Reynolds number in all our experimental configurations are less than 40. In addition, when the particles move near the solid boundary, the wall effects should be taken into account^41,42^. Therefore the applied hydrodynamic force exerted on particles was calculated using the modified Stokes equation^41,43^ (Materials and Methods, Supplementary Figs S1–S4 and Table S1):

2 $${F}_{A}=6\pi a\mu uC$$

where a, µ, u, and C represent the particle radius, the dynamic viscosity of the medium, particle velocity and correction factor. Particle tracking results showed that the particles moved at constant velocity, indicating the particles experienced constant hydrodynamic force throughout the experiment. Since particles move near the channel surface, wall effects should be taken into account using the correction factor (C)

3 $$C={[1-\frac{9}{16}(\frac{d}{2h})+\frac{1}{8}{(\frac{d}{2h})}^{3}-\frac{45}{256}{(\frac{d}{2h})}^{4}-\frac{1}{16}{(\frac{d}{2h})}^{5}]}^{-1}$$

where d is the particle diameter and h is the distance between the particle centroid and the bottom surface (Fig. 2b). The height of the cell ranges from 3 μm to 8 μm^44,45^. Because the variation in correction factor between h = 3 μm and h = 8 μm is negligible (Fig. 2b), we adopted the universal value at h = 5 μm for every estimation. The resulting C was 1.184. It should be noted that the shear stress or friction drag force due to the flow is negligible compared to the effect of pressure drag force exerted on to the particles in our system, given the size of the particles. The drag force (F_D_) at the cell-fluid interface required to be overcome was calculated by the following equation characterizing particles moving at an interface^46^, in this case between the medium and the cytoplasm

4 $${F}_{D}=6\pi a{\mu }_{c}uf$$

where f is the drag coefficient of a translating interfacial particle, and μ_c_ is the assumed viscosity of the protein network underneath the plasma membrane based on our measurement (550 Pa·s, see next section for details). The value of f is a function of the contact angle θ, described by the formula

5 $$f(\theta )=0.5[1+\frac{9}{16}\,{\cos }\,\theta -0.139{co}{{s}}^{2}\theta ]$$

The contact angle θ is defined as the angle between the horizontal line and the line tangetial to the particle surface in contact of the cell/medium interface. The value of θ was determined by the 3D images of cells decorated with CD80-coated particles and stained with membrane dye (Fig. 2c). The particles exhibited 90° ± 5° contact angle in the 10 particles examined using 63X magnification after fixing cells and staining them with the membrane dye. Given the variation in *f*(*θ*) was small (<10°) and the resulting difference in the f value is negligible, *f*(*θ*) was calculated at *θ* = 90° and used for all the estimation. Traction forces in 48 particles were calculated (Fig. 2d). The measured traction force magnitude was 157.6 pN ± 7.2 pN (Fig. 2e, Supplementary Fig. S5). It should be noted that at the timescale of our measurement, the part of the cells in contact with the particles, namely the plasma membrane and the cortical actin network, can be viewed as a viscous fluid^47^. Therefore, Eq. (4)^46^, which corrects for particles moving at fluid interfaces, can be applied.

To compare our method to other force measurement methods, we also performed magnetic tweezers measurement to evaluate the dorsal traction forces transmitted via CTLA4-CD80 bonds, following the previously established protocol^48–50^. The results obtained from the magnetic tweezers showed traction force values exerted through CTLA4-CD80 were of the same order of magnitudes with the results using our microfluidics-based method with no significant difference between the two measurement methods (Supplementary Fig. S6).

**Figure 3:**
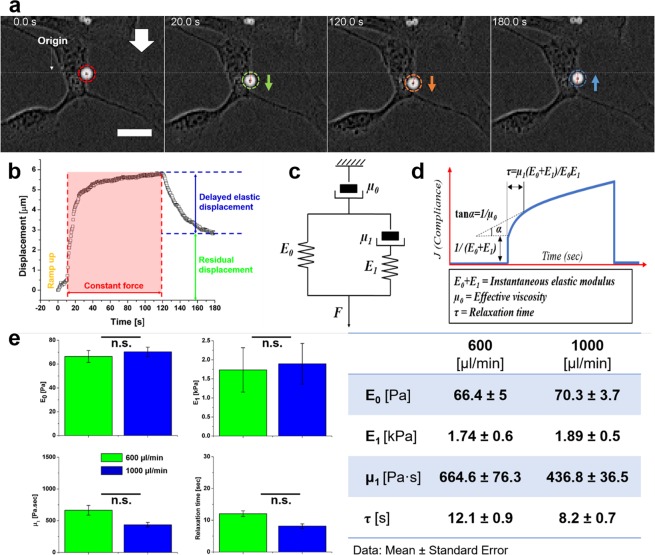
The viscoelasticity of single cells can be measured using flow rates of 600 and 1000 μl/min. (**a**) 2.8-μm particles conjugated with integrin are bound to the surface of the cells. A cell-bound particle is displaced when the flow is on and moves to towards its initial position when the flow is off, exhibiting typical viscoelasticity. The original images were inverted so that the particle appears to be bright and the background dark, for the purpose of better visualization. White arrow indicates flow direction. Scale bar: 20 μm. (**b**) The particle displacement is plotted against time. (**c**,**d**) The typical viscoelastic behavior over time of the cell subjected to deforming forces can be observed in the plot. The Kelvin-Voigt 4 element model used to fit the displacement data consists of two spring elements and two dashpot elements representing the effective elastic modulus and the effective viscosity. (**e**) The viscoelastic parameters, including elastic moduli, E_0_ and E_1_, viscosity µ_1_, and relaxation time τ, were evaluated by fitting the particle displacement under the flow rates of 600 µl/min (n = 53) and 1000 µl/min (n = 80). The values obtained from the two flow rates are comparable with no statistically significant differences. Bar (−): p > 0.05, n.s.: not significant.

Next, we set to demonstrate our technique is suitable for measuring viscoelastic parameters of the cell. Two flow rates, 600 µl/min and 1000 µl/min, were deployed, resulting in hydrodynamic forces of approximately 980 pN and 1.56 nN, which exceeded the cell-generated forces (Fig. 2e) by at least an order of magnitude. Interference by cell-generated forces during the measurement was thereby considered negligible. Upon flow application, anti-integrin antibody-coated particles were observed to be displaced in the flow direction (Supplementary Movie S2). Immediately after the flow was stopped, the particles moved back towards their initial positions (Fig. 3a). The dynamic displacement of the particles represented the responses of the cells to external mechanical perturbations. The displacement curve over time exhibits the typical response belonging to viscoelastic materials, consisting of a viscoelastic regime when a constant force is applied, and a recovery regime where the delayed elastic displacement and residual displacement were superimposed after the force was no longer applied (Fig. 3b). To determine the viscoelasticity of the cells, the displacement curves were fitted to the Kelvin-Voigt 4 element model (Supplementary Fig. S7), represented by two spring elements and two dashpot elements. The effective elastic moduli (E_0_ and E_1_) and the effective viscosity (μ_0_ and μ_1_) with relaxation time (τ) (Fig. 3c,d)^51^ can be determined by

6 $$J(t)=\frac{6\pi aX(t)C}{{F}_{A}}=\frac{1}{{E}_{0}}[1-\frac{{E}_{1}}{{E}_{0}+{E}_{1}}{\exp }(\frac{-t}{{\tau }_{\sigma }})]+\frac{t}{{\mu }_{0}},\,\,\tau =\frac{{\mu }_{1}({E}_{0}+{E}_{1})}{{E}_{0}{E}_{1}}$$

*J*(*t*) represents the compliance over time. The values of E_0_, E_1_, μ_0_, μ_1_ and τ determined using the two flow rates were comparable without statistically significant differences. The results suggest our technique presents a suitable range of flow rates for viscoelasticity measurement in which the results are consistent. The average instant elastic modulus and standard error of E_0_ + E_1_ were 1.80 kPa ± 581 Pa and 1.97 kPa ± 533 Pa for 600 µl/min and 1000 µl/min respectively (Fig. 3e), agreeing with the previously reported measurement (Supplementary Table S2).

Acoustic force spectroscopy was recently developed^52,53^ for viscoelasticity measurement simultaneously in multiple cells, which can apply forces up to 500 pN if 6.84-μm particles are used. In this proof-of-concept report, we chose to use 2.8-μm particles, but our technique, with different choices of bead size, can provide broader range of forces from sub-pN to tens of nN. Passive microrheology, recording dynamic displacements of microparticles without external force perturbation, yields measurement results agreeing with the theoretical predictions in a wide range of biological context. However, passive microrheology requires correct assumption or prior knowledge of the fluids which the particles were immersed in. The rheological properties of the fluids can be controlled in our microfluidic-based method. In addition, passive microrheology requires high spatial resolution to obtain accurate measurement, given the displacement magnitude without external perturbation is subtle. Such requirement can only be fulfilled with microscopy of high magnification and high numerical aperture, which restricts the field of view and subsequently the throughput of the measurement^54^. With the capacity to manipulate the flow rate and subsequent particle displacement using our method, high spatial resolution is a less instrumental restriction in many applications.

**Figure 4:**
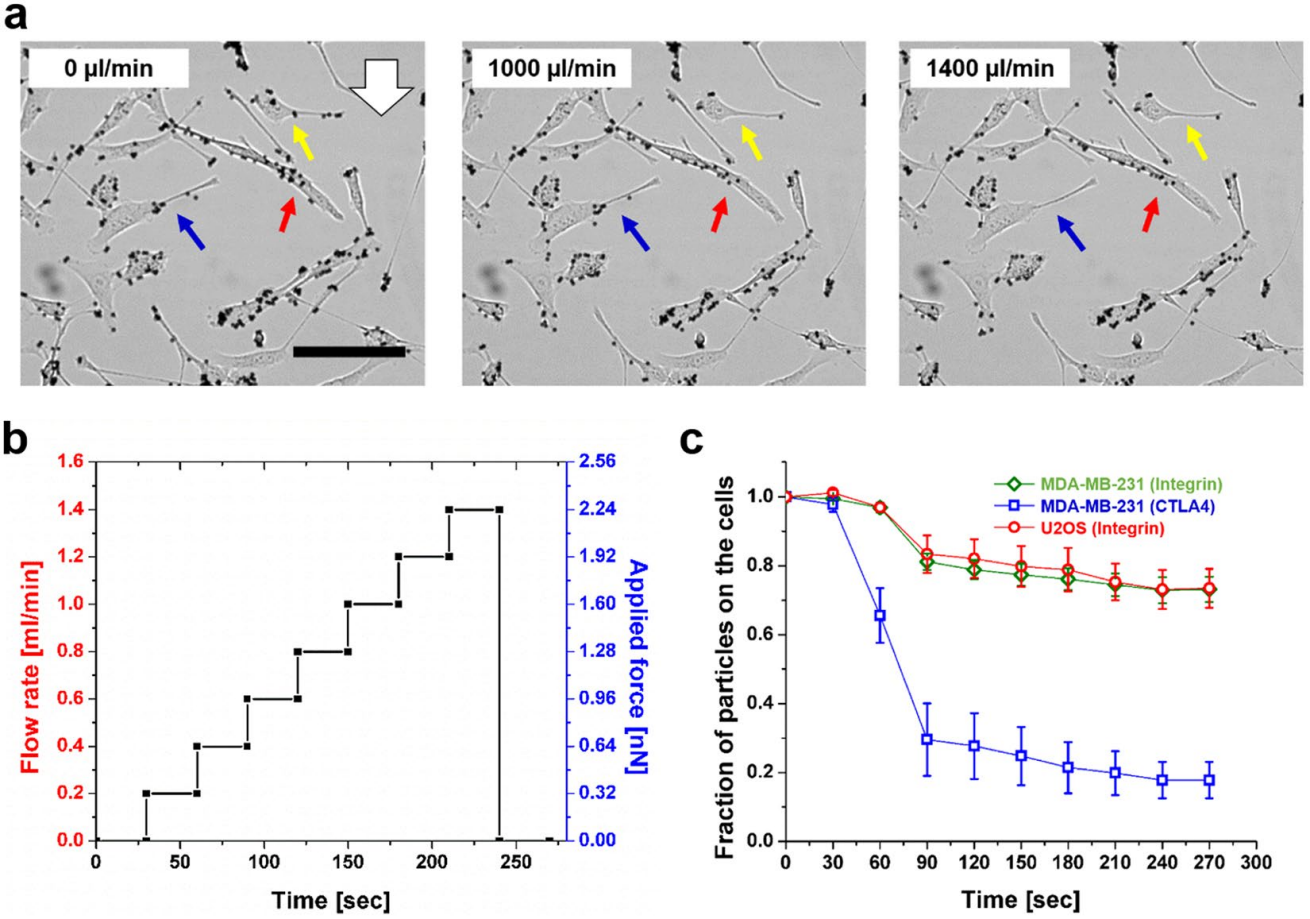
The rupture force between ligand-receptor bond can be measured *in situ* at the dorsal side of the cell using high flow rates. (**a**) Particles increasingly detach from the cells as the flow rate increases. For example, the ligand-receptor bond rupture can be observed in the cells indicated by the yellow, red, and blue arrows. White arrow indicates flow direction. Scale bar: 100 μm. (**b**) Particles bound to the cells are imaged over 270 seconds when subjected to incremental flow rates and the resulting applied forces. The flow rate was increased step-wise (step size = 200 μl/min, 30 seconds/step). (**c**) The number of cell-bound particles decreases as the flow rate and the resulting applied forces increase. The rupture forces of integrin-antibody bond and the CD80-CTLA4 bond are measured. The sharp drop of the particle number is observed in the case of CD80-CTLA4 at 200 µl/min, indicating approximately 320 pN of force is required to disrupt the bond formed between CD80 and CTLA4 at the cell surface. In the case of integrin-antibody, the measurement results from MDA-MB-231 and U2OS cells are consistent, where ~27% of the bond population are disengaged by forces of ~640 pN (400 µl/min).

Next, we deployed a wide range of flow rates to demonstrate the capacity of determining the binding strength between ligands and receptors. Two binding pairs were tested: CD80 vs. CTLA4, and integrin vs. anti-integrin antibody. The particles were coated either by CD80 or anti-integrin antibody. The binding strength between CD80 and CTLA4 was tested in MDA-MB-231 cells, whereas the bond between integrin and anti-integrin was tested in both MDA-MB-231 and osteosarcoma U2OS cells. Incremental flow rates were applied step-wise^55^ with 30-second intervals for a duration of 270 seconds. The rate increment was 200 µl/min per step (Fig. 4b). The number of cell-bound particles decreased as the flow rate increased (Fig. 4a and Supplementary Movie S3).

In the case of CD80-CTLA4 bond, a sharp drop in the number of cell-bound particles was observed when 320-pN hydrodynamic forces were applied (200 µl/min). The bond between integrin and anti-integrin antibody exhibited a distinct pattern (Fig. 4c) from CD80-CTLA4, but the pattern was consistent across cell types. The sharp reduction in cell-bound particles was detected at 640 pN (400 µl/min). However, the number of cell-bound particles did not further decrease even when higher forces were applied, indicating a sub-population of bonds between integrin and anti-integrin antibody exist, where the required rupture force is much higher than the maximal hydrodynamic forces in our platform. Notably, our observation agreed with the previous report by Sheetz and colleagues^56^.

In this study, we present an economical, versatile and high-throughput force measurement technique. It should be noted that the accuracy of traction force estimation depends on the accuracy of the θ value (Fig. 2c). If θ ranges from π/3 to 2π/3, the error can be as large as 33%. Therefore, measurement of θ by 3D imaging is recommended prior to calculation.

Our platform is not intended for single-molecule force measurement. Instead, the particles are coated with high-density ligands (5 × 10^7^ ligands/particle), the rupture and traction forces measured are the products of *in situ* receptor density multiplied by force per molecule. By evaluating the contact area between the particle and the cell surface (Fig. 2c), the dorsal traction force generated per unit area can be estimated. Similarly, the force per unit area required to break two cells adherent to each other through specific ligand-receptor binding can be calculated. Our technique thereby is relevant in physiological scenarios and can be applied, for example, to evaluate forces required to disrupt cancer cell-endothelium association via ICAM-MUC1^57^ bond before intrastation is initiated, or to evaluate the pulling force, transmitted through Notch and its ligands at the cell-cell interface during embryonic development^58^.

## Materials and Methods

### Cell culture and reagents

The immortalized human breast carcinoma cell line MDA-MB-231 and human osteosarcoma cell line U2OS were maintained in culture medium (Dulbecco’s Modified Eagle’s Medium (ThermoFisher 11995073) for MDA-MB-231 and McCoy’s 5A Medium (Sigma-Aldrich M8403) for U2OS. The media were supplemented with 100 U/mL penicillin and 100 μg/mL streptomycin (1% v/v P/S) (ThermoFisher 15140122), and 10% (v/v) fetal bovine serum (ThermoFisher 26140079). The cells were cultured in 10 ml of supplemented culture medium in 10 cm diameter cell culture dish (Nest Scientific 704001) at 37 °C and 5% CO_2_. Cells were collected using 0.25% trypsin and 2.21 mM EDTA (Corning Cellgro 25-053-CI).

### Cell preparation in a microfluidic channel

Approximately 50,000 cells, with the density of 2,500,000 cells/ml, were seeded into a microfluidic channel (width (*w*) × height (*h*) = 1 mm × 100 μm) coated with Poly-L-lysine, Poly-D-lysine, fibronectin, and collagen (µ-Slide VI 0.1, Ibidi, 80666). The microfluidic device was placed in an incubator (5% CO_2_ and 37 °C) for about 24 hours until cells adhered to the surface with a well-spread morphology.

### Imaging and image processing

The time-lapse images were acquired using the optical microscope (Leica SP8) equipped with a CCD camera (Leica DFC365FX) and a 10X objective (NA 0.45). The frame rate of each time-lapse image series were set as indicated in each measurement procedure in the following. The 3D confocal images of particles on cells stained with membrane dye were performed using Leica SP8 with white light laser and photomultipliers. The z-sectioning of the 3D images was set at 0.01-μm intervals for 3D reconstruction based on optimized 3D deconvolution using Hyugens Software (Scientific Volume Imaging). All images were processed and analyzed using FIJI-ImageJ (NIH) and MATLAB (R2018a, MathWorks).

### Particle coating

To fabricate CD80- and anti-integrin α5 antibody-coated particles, we conjugated recombinant CD80-Fc (Biolegend 555404) or anti-integrin α5 antibody (Biolegend 328002) onto 2.8-µm Dynabeads (ThermoFischer 10003D) via Protein G. Dynabeads and CD80-Fc or anti-integrin α5 antibody, as mixed at the ratio of 1:5 (protein G: ligand). The mixture was incubated at room temperature while rotating at 30 rpm for 1 hour. The mixture was then washed for 3 times with PBS to exclude the free CD80 or anti-integrin α5 antibody in the buffer.

### Membrane staining of the cells

Cells with CD80- or anti-integrin α5 antibody-coated particles bound to their surfaces were subject to the membrane dye staining to determine the interfacial contact angle between the cell and the medium as mentioned in the main text. The cells were incubated in the 1000X-diluted CellMask™ plasma membrane dye (ThermoFischer C10046) in the appropriate medium at 37 °C for 5 minutes. The cells were then washed for 3 times with PBS before fixation by 4% paraformaldehyde. The fixed cells were then imaged using the Leica SP8 confocal microscope.

### Microfluidic channel configurations for force measurement

CD80- and anti-integrin α5 antibody-coated particles were added into the microfluidic channel following the protocol provided by the manufacturer (Ibdi). After incubation for 5 minutes at 37 °C and 5% CO_2_, the microfluidic channel was connected with a syringe pump (NE-1002X, Pump System Inc.), a syringe (60 mL, SMP Medical), silicone tubing (Ibidi 0841), and an elbow luer connector (Ibidi 10802). The channel was then mounted on the microscope.

### Measurement of dorsal traction forces

A constant flow rate of 10 μl/min resulting in 14 pN as the maximal particle drag force was utilized to create hydrodynamic forces. The MDA-MB-231 cells decorated with the CD80-coated particles were imaged at 10 s interval for 5 minutes. The particle coordinates were tracked to measure displacement using TrackMate (FIJI-ImageJ, NIH), and subsequently used to evaluate drag forces at the interface experienced by the particles. The drag force at the interface of the particle was calculated by the equation assuming the interfacial particle at the fluid-fluid interface as described in the main text^59^. The dorsal traction force magnitudes were then calculated by adding the magnitudes of the applied force and the drag force at the interface. Five independent experiments were performed. Only non-aggregated single particles were selected for analysis.

### Measurement of viscoelastic properties of the cell

We deployed two flow rates, 600 μl/min, and 1000 μl/min, to apply different forces on the anti-integrin-conjugated particles on MDA-MB-231 cells. The cells were imaged at 0.5 s interval for 3 minutes. The particle coordinates were tracked to measure displacement using TrackMate, and used for the assessment of the viscoelastic properties of the cells. Five independent experiments were performed. Only non-aggregated single particles were selected for analysis.

### Measurement of the molecular bond strength

The step-wise flow rates ranging from 200 μl/min to 1400 μl/min, with the 200 μl/min increment, were applied to the channel to generate incremental forces exerted on the particles. Three groups, anti-integrin α5 antibody /integrin on MDA-MB-231 cells, anti-integrin α5 antibody /integrin on U2OS cells, and CD80/CTLA4 on MDA-MB-231 cells, were used for the measurement. The cells were imaged at 1 s interval for 4.5 minutes. The particle coordinates were tracked by TrackMate and used for the bond strength estimation. Three independent experiments were performed.

### Estimation of the number of ligands conjugated to the particle

According to the data provided by the manufacturer, 1 mg of Dynabeads-protein G binds ~8 µg of human IgG. Given that the density of the Dynabeads is ~1.3 g/ml, 1 mg of Dynabeads equates to 6.09 × 10^8^ particles. Given that the molecular weight of IgG is approximately 150 g/mole, 8 µg of IgG equates to 3.2 × 10^16^ molecules. Because we mixed the ligands and particles at the ratio of 1:5 (protein G: ligand), and protein G has high affinity to Fc-CD80 or IgG, resulting in saturation of the binding sites on the particle, it is estimated that each particle is conjugated with 5 × 10^7^ ligands on its surface.

### Measurement of traction forces using magnetic tweezers

The 2.8-µm, anti-CTLA4 paramagnetic particles were added to bind the surface of the cell. The 416-steel pole tip powerd by Neodymium magnets (0.4-T surface field, K&J Magnets) was then positioned close to a paramagnetic particle bound to the cell. The particle motion induced by the magnetic field gradient was tracked over time. To calibrate the force field, unbound, free-moving particles in the image field were tracked. The magnetic forces were assumed equal to the drag forces experienced by the particle, which could be calculated by the modified Stokes equation. The dorsal traction was calculated based on the equation

7 $${F}_{C}={F}_{M}+{F}_{D}$$

where F_C_, F_M_, and F_D_ are the traction force generated by cell, applied magnetic force, and drag forces at the interface, respectively.

### Numerical analyses of fluid flow and particle transport

We applied the finite element/volume method (FEM/FVM) to simulate fluid and particle dynamics available in ANSYS (version 14.5). The flow motion can be described by solving two classical equations, conservation of mass and conservation of momentum (Navier-Stokes equation), with the assumption of steady-state and incompressible flow given by^60,61^:

8 $${\nabla }\cdot {\boldsymbol{V}}={\rm{0}}$$

9 $$-{\nabla }p+\mu {{\nabla }}^{2}{\boldsymbol{V}}=\rho ({\boldsymbol{V}}\cdot {\nabla }){\boldsymbol{V}}$$

where **V** represents the fluid velocity vector and *p* the fluid pressure, µ the dynamic viscosity and ρ the water density.

Total 6 particles with 2.8-µm diameter were used in the simulation. The simulation was initiated by injecting particles into the inlet of the microfluidic channel with 1-mm width and 100-µm height. The particles were placed at varied locations in terms of distances to the bottom surface, ranging from 25 µm to 500 µm (Fig. S1). For the boundary conditions, constant flow rates of 10 µl/min, 50 µl/min, 100 µl/min, 300 µl/min, 600 µl/min, and 1000 µl/min, and the pressure of 0 Pa were set as the inlet and outlet conditions. The wall was assumed to be non-slip. The flow is laminar and Reynolds number ranges from 0.27 to 40 depending on the flow rate. For particle dynamics, particle transport equation was used^62^.

10 $$m\frac{d{{\boldsymbol{V}}}_{{\boldsymbol{p}}}}{dt}=\frac{1}{8}\pi {\rho }_{f}{d}_{p}^{2}{C}_{D}|{{\boldsymbol{V}}}_{{\boldsymbol{f}}}-{{\boldsymbol{V}}}_{{\boldsymbol{p}}}|({{\boldsymbol{V}}}_{{\boldsymbol{f}}}-{{\boldsymbol{V}}}_{{\boldsymbol{p}}})$$

where *m*, *d*_*p*_, **V**_p_, *ρ*_*f*_, *C*_*D*,_ and **V**_f_ are the particle mass, particle diameter, particle velocity, fluid density, particle drag coefficient, and fluid velocity.

Drag coefficient, C_D_, can be calculated using the Schiller-Naumann correlation as follows^63^:

11 $${C}_{D}=\frac{24}{R{e}_{p}}(1+0.15\,R{e}_{p}^{0.687})$$

where Re_p_ is the particle Reynolds number.

The hexahedral mesh was created using ANSYS ICEM-CFD. The number of elements and nodes were 140,679 and 150,000, respectively. The specialized program for simulation of fluid dynamics, ANSYS-CFX, was used for the computation. The root mean square (RMS) residuals were utilized to set convergence criteria as 10^−4^–10^−5^ ^64^.

### Calculation of the applied drag forces exerted on cells

The simulation showed that particles travel in the microfluidic channel in a smoothly continuous manner (Fig. S2). The traveling time and velocity of the particles were then quantified. It reveals that particles are slower when closer to the channel wall, due to the non-slip condition, resulting in longer traveling time near the wall compared to near the center (Fig. S3). Individual particle velocity is constant throughout the fully developed region of the channel. Particle drag forces were calculated using the modified Stokes equation as shown in Eq. (2)^41,43^. The drag force decreases near the wall as it is proportional to the particle velocity (Fig. S4). The calculated drag force (squares in Fig. S4) for each flow rate was non-linearly fitted to the logistic model given by (R^2^ > 0.95):

12 $$F=\frac{{A}_{1}-{A}_{2}}{1+{(x/{x}_{0})}^{p}}+{A}_{2}$$

The 4 parameters (A_1_, A_2_, x_0_, and p) acquired from the logistic model are shown in Table S1. To avoid the wall effects imposed by the side walls of the channel, only cells at locations more than 200 μm away from the side walls were analyzed.

### Calculation of applied hydrodynamic forces exerted on particles

Given that the applied hydrodynamic force, or known as applied drag force, is a function of the distance from the wall/surface, the particle location was taken into account during the calculation. The hydrodynamic force can be computed by substituting the value of the distance into Eq. (13). To avoid the wall effects imposed by the side walls of the channel, only cells at locations more than 200 μm away from the side walls were analyzed.

### Calculation of shear modulus, elastic modulus, and viscosity of the cell

After the dynamic displacement of each particle was determined, it was fit to the Kelvin-Voigt 4 element model^51^:

13 $$J(t)=\frac{6\pi aX(t)C}{{F}_{A}}=\frac{1}{{G}_{0}}[1-\frac{{G}_{1}}{{G}_{0}+{G}_{1}}{\exp }(\frac{-t}{\tau })]+\frac{t}{{\mu ^{\prime} }_{1}},\,\,\tau =\frac{{\mu ^{\prime} }_{1}({G}_{0}+{G}_{1})}{{G}_{0}{G}_{1}}$$

where *G*_0_ and *G*_1_ are the shear modulus, and *µ*′_0_ and *µ*′_1_ are viscosity.

The calculated shear modulus and viscosity can be converted into the elastic modulus and the modified viscosity in the main text using the following form:

14 $$\{\begin{array}{c}{E}_{i}=2{G}_{i}(1+\nu )\\ {\mu }_{i}=2{{\mu }^{{\rm{^{\prime} }}}}_{i}(1+\nu )\end{array}$$

where the index, *i*, is 0 and 1. A Poisson ratio has been experimentally measured ranging from 0.4 to 0.5^65^. Here we used 0.4 as the Poisson ratio. To avoid the wall effects imposed by the side walls of the channel, only cells at locations more than 200 μm away from the side walls were analyzed.

### Statistical analysis

The data in this study were statistically analyzed using the Student’s *t*-test for paired data (two-tail). P values less than 0.05 were taken into account statistically significant. Data are presented as mean ± standard error.

## Supplementary information

Movie S1

Movie S2

Movie S3

Supplementary Materials
