# Supplementary material for: Versatile and High-throughput Force Measurement Platform for Dorsal Cell Mechanics
Source: Sci Rep. 2019 Sep 16;9:13286. doi: 10.1038/s41598-019-49592-1 (PMC6746792; doi:10.1038/s41598-019-49592-1)
Supplement: Supplementary file 4 — Supplementary Materials [file 41598_2019_49592_MOESM4_ESM.docx]

Versatile and High-throughput Force Measurement Platform for Dorsal Cell Mechanics

Seungman Park^1^, Yoon Ki Joo^2^, Yun Chen^1,3,4*^

^1^ *Department of Mechanical Engineering, Johns Hopkins University, MD, USA*

^2^ *Department of Chemical and Biomolecular Engineering, Johns Hopkins University, MD, USA*

^3^ *Center for Cell Dynamics, Johns Hopkins University, MD, USA*

^4^ *Institute for NanoBio Technology, Johns Hopkins University, MD, USA*

^*^Corresponding Author: Yun Chen

Department of Mechanical Engineering

Johns Hopkins University

Email: yun.chen@jhu.edu

Phone: +1-410-516-5194

**Supplementary Figures**


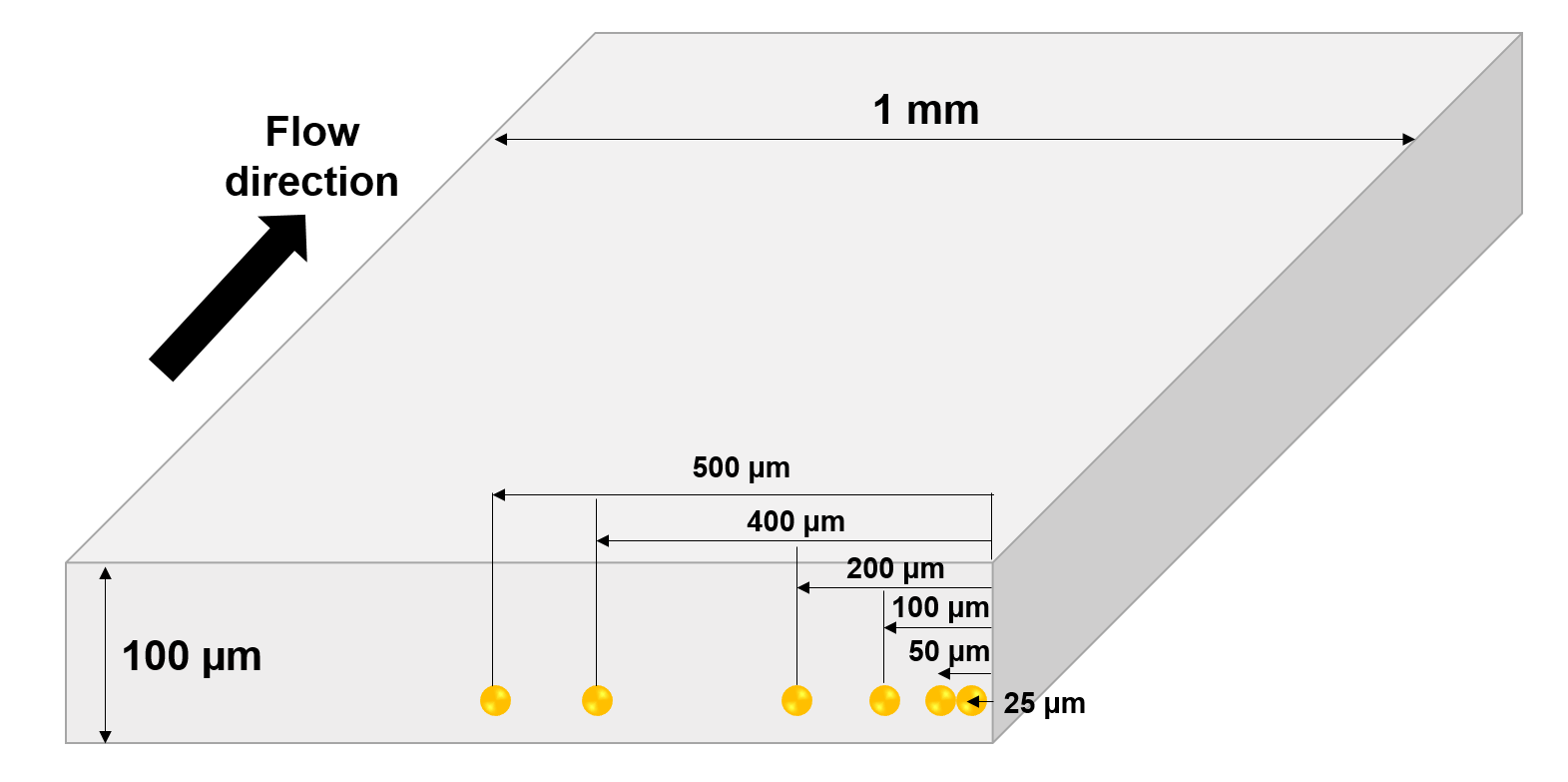


Fig. S1. The hydrodynamic force is calculated by simulating 2.8-µm particles moving along the microfluidic channel with 1-mm width and 100-µm height. In the simulation, the particles were positioned near the inlet for 6 different locations from the bottom surface of the channel. Different flow rates were applied at the inlet as the boundary condition.


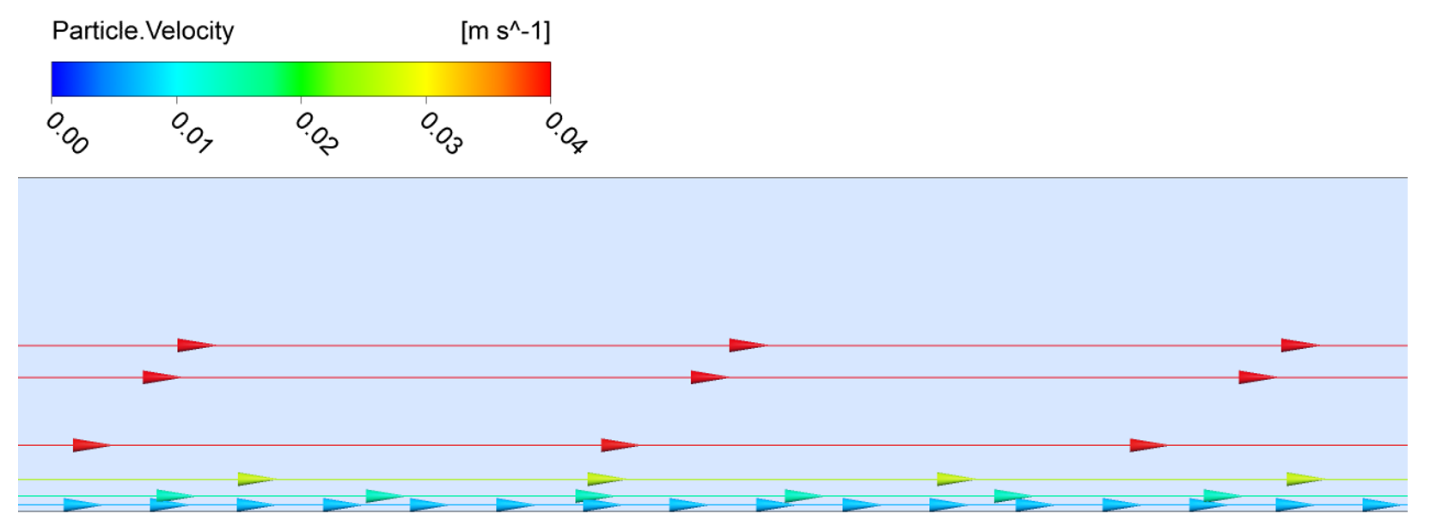


Fig. S2. Simulated particle path lines for the varied distance from the wall in the microfluidic channel are shown (top view). Each line shown in the figure indicates the path line for each particle positioned at varied distance from the surface as it passes through the microfluidic channel. The color of each line indicates the velocity. The simulated velocity at the varied distance is then used to calculate the drag force exerted on the particle by the flow.


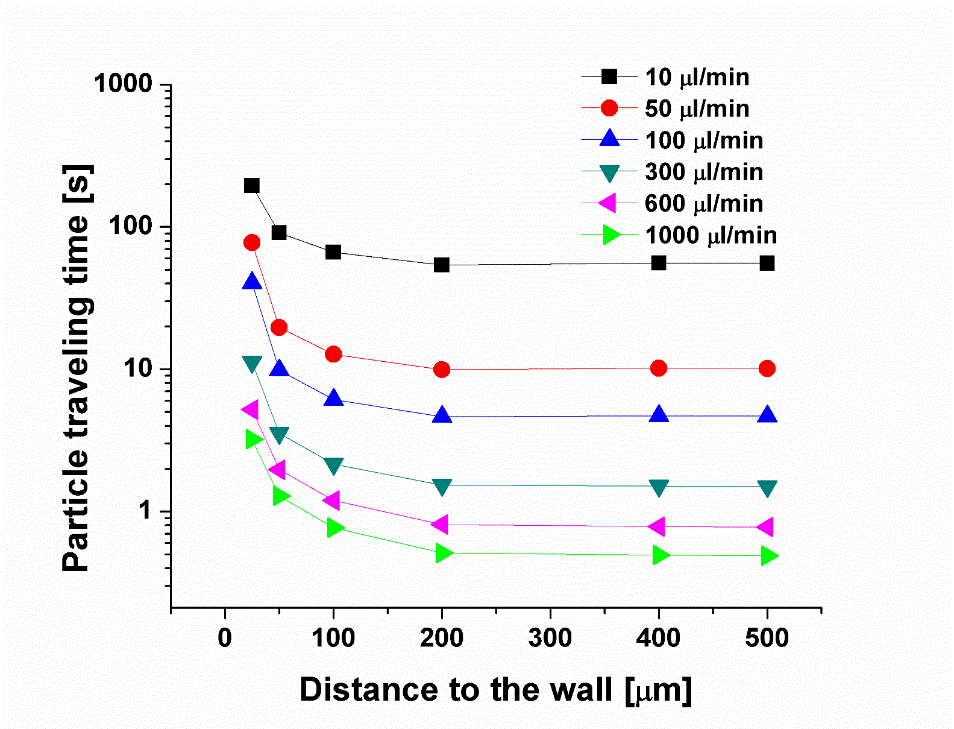


Fig. S3. The simulation shows the trend that when the distance between the particle and the wall is less than 200 µm, the closer the particles are from the bottom surface, the slower they move, requiring more time to travel through the microchannel. For distances larger than 200 µm from the wall, traveling time remains almost constant, indicating constant velocity and force.


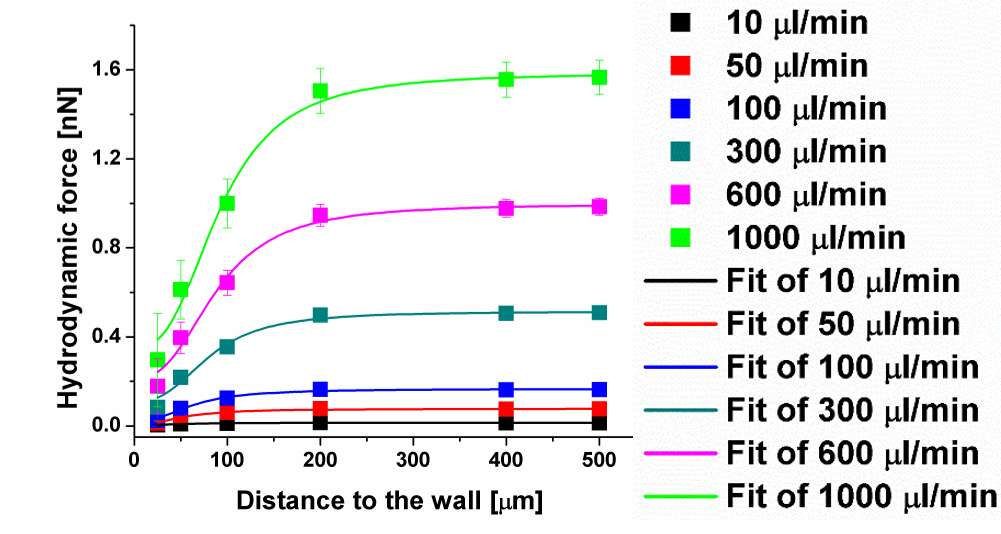


Fig. S4. Different flow rates result in different magnitudes of hydrodynamic forces. The higher the flow rate, the higher the force. The force sharply decreases closer to the wall. R^2^ > 0.95 for fitted data.





Fig. S5. 2.8-µm particles conjugated with CD80 on MDA-MB-231 cells were tracked, and the magnitude distribution of the drag force at the interface (or net force) is shown in the histogram with an average of 143.6 pN and median of 140.1 pN (n=48).


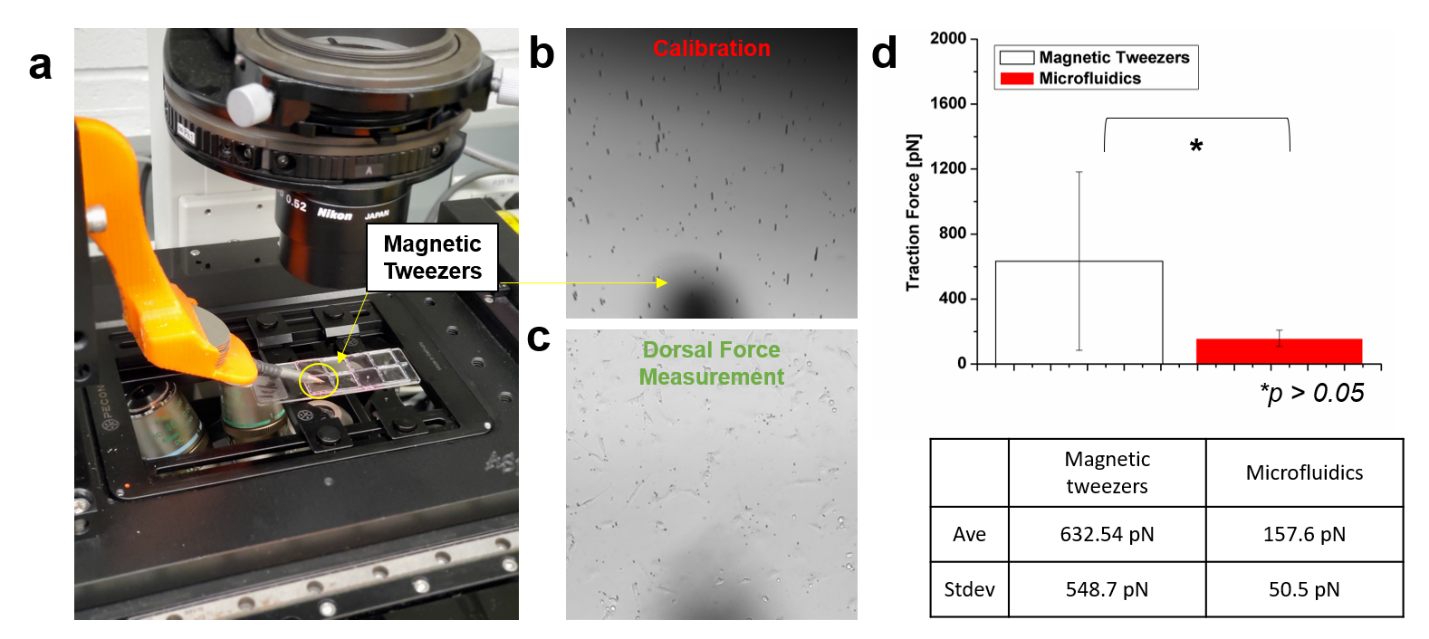


Fig. S6. Validation for dorsal traction force measurement results using magnetic tweezers. **a**. Nneodymium magnetic pole tip was positioned near magnetic beads (d = 2.8 µm) bound to the cell surface and to apply the magnetic forces. **b,c**. The displacement if the particle over time was tracked in the absence and the presence of the cells to calibrate the magnetic force magnitudes as a function of the distance from the pole tip (**b**) and dorsal traction force magnitudes (**c**) were measured to track. **d**. The dorsal traction forces measured by the magnetic tweezers (n=8) are statistically comparable to the values obtained by the microfluidic-based, though with a higher standard deviation **p*>0.05

**
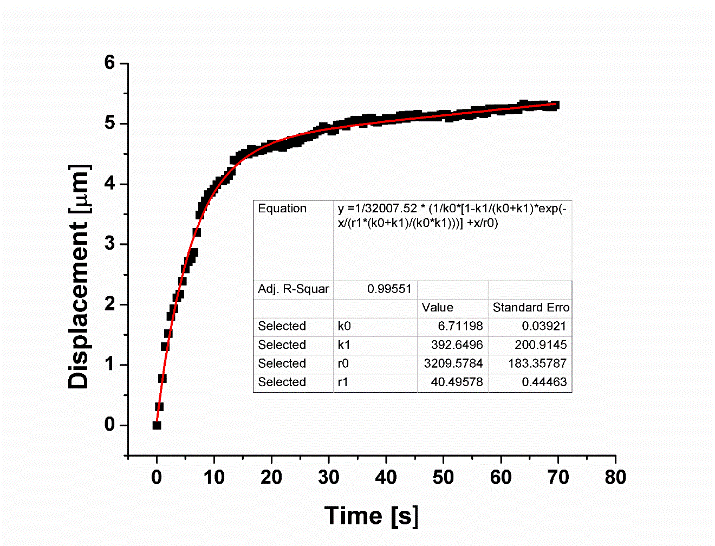
**

Fig. S7. The displacement curve is fitted using the Kelvin-Voigt 4 element model to estimate the elastic moduli, viscosity coefficient and relaxation time of the cell ^1,2^. k0 and k1 represent the elastic moduli, and r0 and r1 represent the effective viscosity values.

**Supplementary Tables**

Table S1: Parametric values obtained from the fitting (Fig. S4) for each flow rate.

|  | **10 [µl/min]** | **50** | **100** | **300** | **600** | **1000** |
| --- | --- | --- | --- | --- | --- | --- |
| **A_1_** | 1.30E-12 | -5.02E-13 | 1.70E-11 | 1.11E-10 | 2.17E-10 | 3.54E-10 |
| **A_2_** | 1.42E-11 | 7.76E-11 | 1.66E-10 | 5.14E-10 | 9.97E-10 | 1.59E-09 |
| **x_0_** | 4.53E-05 | 5.07E-05 | 6.16E-05 | 8.12E-05 | 8.86E-05 | 9.19E-05 |
| **p** | 1.89403 | 2.02424 | 2.2997 | 2.70643 | 2.66731 | 2.7478 |

Table S2: Elastic moduli and/or viscosity values reported by previous studies

| **Cell type** | **Technique** | **Elastic modulus** | **Viscosity** | **Author** |
| --- | --- | --- | --- | --- |
| **Kidney cancer cell** | AFM | 0.5 - 1.1 kPa | 20 - 60 Pa.s | Rebelo ^3^ |
| **NIH-3T3**  **NMuMG**  **MDA-MB-231**  **MCF-7** | AFM | 2.884 kPa  2.971 kPa  1.918 kPa  1.292 kPa |  | Efremov ^4^ |
| **Neutrophil**  **Monocyte**  **Macrophage** | Optical tweezers |  | ~ 100 Pa.s | Ekpenyong ^5^ |
| **NIH-3T3** | Microfluidics | G = ~ 8 kPa |  | Guillou ^6^ |
| **B-lymphocytes** | AFM | 2 kPa |  | Li ^7^ |
| **ATDC5 cell**  **NIH-3T3** | Acoustic tweezers | 331 MPa  118 MPa |  | Yang ^8^ |

**Supplementary Movies**

Movie S1: Representative movie of dynamic particle movement in the dorsal traction forces measurement. Pink arrow indicates flow direction.

Movie S2: Representative movie of dynamic particle movement and displacement in the viscoelasticity measurement. Please note that the original movies were inverted so that the particle appears to be bright and the background dark, for the purpose of better visualization. Pink arrow indicates flow direction.

Movie S3: Representative movie of dynamic particle movement and fraction of detachment on cells in the bond strength measurement. Pink arrow indicates flow direction.

**References**

1. Bausch, A. R., Ziemann, F., Boulbitch, A. A., Jacobson, K. & Sackmann, E. Local measurements of viscoelastic parameters of adherent cell surfaces by magnetic bead microrheometry. *Biophys. J.* **75**, 2038–2049 (1998).

2. Park, S. *et al.* Mechanical Characterization of hiPSC‐Derived Cardiac Tissues for Quality Control. *Adv. Biosyst.* **2**, 1800251 (2018).

3. Rebelo, L. M., De Sousa, J. S., Mendes Filho, J. & Radmacher, M. Comparison of the viscoelastic properties of cells from different kidney cancer phenotypes measured with atomic force microscopy. *Nanotechnology* **24**, (2013).

4. Efremov, Y. M., Wang, W. H., Hardy, S. D., Geahlen, R. L. & Raman, A. Measuring nanoscale viscoelastic parameters of cells directly from AFM force-displacement curves. *Sci. Rep.* **7**, (2017).

5. Ekpenyong, A. E. *et al.* Viscoelastic Properties of Differentiating Blood Cells Are Fate- and Function-Dependent. *PLoS One* **7**, (2012).

6. Guillou, L. *et al.* Measuring Cell Viscoelastic Properties Using a Microfluidic Extensional Flow Device. *Biophys. J.* **111**, 2039–2050 (2016).

7. Li, M., Liu, L., Xi, N. & Wang, Y. Atomic force microscopy studies on cellular elastic and viscoelastic properties. *Sci. China Life Sci.* **61**, (2018).

8. Yang, C., Chen, D. & Hong, X. Estimation of Viscoelastic Properties of Cells Using Acoustic Tweezing Cytometry. *J. Ultrasound Med.* **35**, 2537–2542 (2016).
